# The messy process of guiding proteins into membranes

**DOI:** 10.7554/eLife.12100

**Published:** 2015-11-06

**Authors:** Stephen H White

**Affiliations:** Department of Physiology & Biophysics, University of California, Irvine, Irvine, United Statesshwhite@uci.edu

**Keywords:** membrane protein topology, simulation, dual-topology, topogenesis, none

## Abstract

A new simulation protocol has revealed unexpected complexity in the folding of membrane proteins.

**Related research article** Van Lehn RC, Zhang B, Miller TF. 2015. Regulation of multispanning membrane protein topology via post-translational annealing. *eLife* **4**:e08697. doi: 10.7554/eLife.08697

**Image** Membrane proteins may fold into their final shape after they have been inserted into the membrane

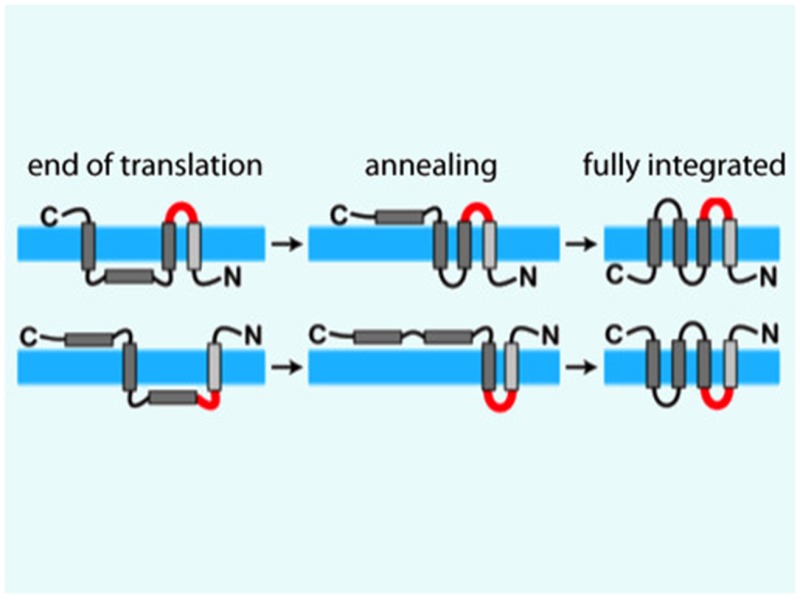

One of the keys to predicting the three-dimensional structure of a membrane protein from its sequence of amino acid residues is to understand how structures called translocons guide the protein to its final folded state. Translocons are generally thought of as channels that allow proteins to cross cell membranes. In eukaryotes, it is thought that newly-formed secreted proteins pass through the Sec61 translocon as they emerge from the ribosome. New membrane proteins are thought to follow a similar path, except that the hydrophobic transmembrane helices in these proteins are diverted sideways so that they become embedded in the cell membrane. This ‘sequential-insertion’ scheme seems logical in the context of what we know about the structure of translocons (Rapoport et al., 2004; Cymer et al., 2015), but is it correct?

We cannot answer this question because we do not have experimental methods that can follow, residue-by-residue, the insertion and folding of the protein chains as they pass from the ribosome and into the membrane. The alternative is to simulate the process. However, a newly-formed protein chain elongates at a rate of about one residue every 50–100 milliseconds, which is orders of magnitude faster than can be modeled using standard molecular dynamics simulation methods. Now, in eLife, Reid van Lehn, Bin Zhang and Thomas Miller of the California Institute of Technology report a simplified approach that allows insertion and folding to be simulated on biological time scales (Van Lehn et al., 2015). Their results suggest that the membrane protein insertion/folding process is more complicated than commonly depicted in the sequential-insertion scheme.

Van Lehn et al. modeled a protein called EmrE that sits in the inner membrane of *Escherichia coli* bacteria and is able to transport a wide range of antibiotic drugs out of the cell. This helps to make the bacteria resistant to these treatments. EmrE is a homodimer, and each monomer has four transmembrane helices (Chen et al., 2007). EmrE is unusual in that the two monomers are oriented in opposite directions (Figure 1A): this is known as dual topology.

**Figure 1.**
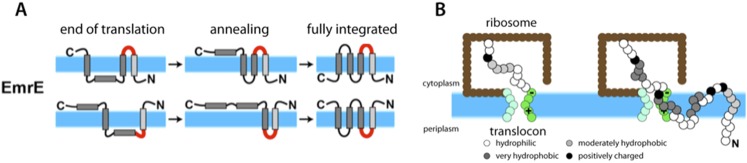
Simulations suggest that membrane proteins take on their final structure after they have been inserted into the membrane. (**A**) The topologies of the EmrE monomers first inserted into the cytoplasmic membrane (blue band) at the end of translation (left) do not necessarily reflect the final topologies, which are subsequently achieved through thermodynamics-driven annealing. The interhelical loops in red represent the loops that flip most slowly, and thereby have a major influence on the kinetics of folding. EmrE can take on two different, antiparallel topologies; each row in the figure shows how one of these topologies may develop. (**B**) Van Lehn et al. used a coarse-grained model to simulate the insertion and folding of the EmrE dual-topology membrane protein (Zhang and Miller, 2012). Coarse-grained beads are assigned approximate hydrophobicity values (indicated by the shadings of the beads). The ribosome (brown) and translocon (green) are also represented as coarse-grained beads. The translocon is negatively charged on the cytoplasmic end and positively charged at the periplasmic end to represent the known charge distribution of the Sec 61 translocon (Goder et al., 2004). The simulation proceeds by adding a bead at the C-terminus of the nascent chain every 125 milliseconds; the panel on the right shows the chain on the left at a later point in time. Figure adapted from Figures 1 and 4 of Van Lehn et al. (2015).

The topology (orientation) of membrane proteins is largely determined by the positive-inside rule (von Heijne, 1986). This rule suggests that if the connecting loops that join the transmembrane regions of the protein are rich in lysine and arginine residues, then these loops tend to orient inward, toward the cytoplasm of the cell. This is known as the K+R bias. EmrE, which is encoded in a single gene, has a weak K+R bias, and this means that the monomers can be inserted into the membrane in one of two opposite orientations (Rapp et al., 2006, 2007).

In 2010, researchers at Stockholm University reported, based on extensive mutation studies, that a single positively charged residue placed in different positions throughout the protein can control the topology of EmrE monomers and affect whether parallel or anti-parallel dimers form (Seppälä et al., 2010). Given the positive-inside rule and the sequential-insertion scheme, one would expect positive charges in the C-terminal region of a membrane protein to have a smaller influence on topology than charges in the N-terminal region. However, Seppälä et al*.* discovered that a single positive charge at the C-terminus itself could determine the orientation of EmrE!

Because the positive-inside rule was robustly verified in the Stockholm experiments, a logical conclusion is that the sequential-insertion scheme does not describe accurately how EmrE, and perhaps other membrane proteins, fold inside cells. The simulations now performed by Van Lehn et al. divulge the missing ingredients of membrane protein folding: stochastic insertion and post-insertion annealing. By stochastic insertion, I mean that protein chains can have various topologies after they have been made, creating what Van Lehn et al. refer to as an ‘end-of-translation ensemble’ (Figure 1A). After being inserted into the membrane, the members of the ensemble that are not initially in their lowest thermodynamic free energy state subsequently relax to their preferred topology through a process called annealing. In the case of EmrE, antiparallel dimers can form because there are two final topologies that have similar free energies.

Van Lehn et al. increased the speed of the simulations by treating the nascent protein chain as a sequence of coarse-grained beads, with each bead representing several amino acids (Figure 1B). Four beads were used to represent the transmembrane helices and five beads were to used represent the loops that connect these helices. Certain properties of the amino acid residues that are known to affect the topology of a protein were also incorporated into the simulation: for example, hydrophobicities were assigned to the beads using an experimentally-determined hydrophobicity scale (Wimley et al., 1996). Particularly important was the assignment of positive charges in the connecting loops between the transmembrane helices to mimic the mutation experiments of Seppälä et al*.* (2010). The ribosome and translocon were also represented by simple two-dimensional structures composed of coarse-grained beads (Zhang and Miller, 2012; Figure 1B). Crucially, the model translocon used in the simulations had two negative charges on its cytoplasmic side and two positive charges on its periplasmic side to mimic the known net charge distribution of the translocon (Goder et al., 2004).

The simulations were performed by adding a new bead at the C-terminal of the nascent chain every 125 milliseconds. In this way, van Lehn et al. simulated the insertion and folding of the many mutant EmrE proteins studied by Seppälä et al. (2010) and found remarkable agreement with the experimentally determined topologies.

The simulations of van Lehn et al. show that the stochastic insertion of newly-formed protein chains into the membrane, followed by thermodynamics-driven annealing, is a viable alternative to the current sequential-insertion view. What is needed now is direct experimental verification of how transmembrane proteins are inserted into the membrane. This will require new methods that can directly follow insertion and folding on the biological time scale.

## References

[bib1] Chen YJ, Pornillos O, Lieu S, Ma C, Chen AP, Chang G (2007). X-ray structure of EmrE supports dual topology model. Proceedings of the National Academy of Sciences of USA.

[bib2] Cymer F, von Heijne G, White SH (2015). Mechanisms of integral membrane protein insertion and folding. Journal of Molecular Biology.

[bib3] Goder V, Junne T, Spiess M (2004). Sec61p contributes to signal sequence orientation according to the positive-inside rule. Molecular Biology of the Cell.

[bib4] Rapoport TA, Goder V, Heinrich SU, Matlack KE (2004). Membrane-protein integration and the role of the translocation channel. Trends in Cell Biology.

[bib5] Rapp M, Granseth E, Seppälä S, Von Heijne G (2006). Identification and evolution of dual-topology membrane proteins. Nature Structural & Molecular Biology.

[bib6] Rapp M, Seppälä S, Granseth E, von Heijne G (2007). Emulating membrane protein evolution by rational design. Science.

[bib7] Seppälä S, Slusky JS, Lloris-Garcerá P, Rapp M, von Heijne G (2010). Control of membrane protein topology by a single C-terminal residue. Science.

[bib8] Van Lehn RC, Zhang B, Miller TF (2015). Regulation of multispanning membrane protein topology via post-translational annealing. eLife.

[bib9] von Heijne G (1986). The distribution of positively charged residues in bacterial inner membrane proteins correllates with the trans-membrane topology. EMBO Journal.

[bib10] Wimley WC, Creamer TP, White SH (1996). Solvation energies of amino acid sidechains and backbone in a family of host-guest pentapeptides. Biochemistry.

[bib11] Zhang B, Miller TF (2012). Long-timescale dynamics and regulation of Sec-facilitated protein translocation. Cell Reports.

